# Supplementary material for: Suggestions for improving the visualization of magnetic resonance spectroscopy voxels and spectra
Source: R Soc Open Sci. 2020 Aug 5;7(8):200600. doi: 10.1098/rsos.200600 (PMC7481722; doi:10.1098/rsos.200600)
Supplement: Appraised papers and scoring [file rsos200600supp1.pdf]

Study	Groups	Voxel location				Spectral quality	
		Location figure	Participants shown	Text description	Voxel size	Spectra figure	Participants shown
Aoki 2015	ASD (Oxytocin treatment)	Drawn	N/A	3	Dimensions	From data	Single
Atagun 2018	SCZ + BD-I + BD-II + HC	Drawn	N/A	2	Dimensions	From data	Single
Atagun 2017	BD-I + BD-II + HC	None	N/A	2	Dimensions	None	N/A
Atagun 2015	SCZ + BD-I + HC	Drawn	N/A	2	Dimensions	From data	Single
Bae 2016	ADHD + HC	Drawn	Single	3	Dimensions	From data	Single
Bauer 2016	ADHD + HC	Drawn	N/A	3	Dimensions	From data	Single
Bollmann 2015	ADHD + HC	Drawn	N/A	1	Dimensions	From data	Single
Bond 2016	BD + HC	Drawn	N/A	1	Dimensions	From data	Single
Bossong 2019	High-risk psychosis + HC	Drawn	N/A	1	Dimensions	From data	Single
Brennan 2017	MDD (Citalopram treatment)	Drawn	N/A	3	Dimensions	From data	Single
Bustillo 2016	SCZ	Drawn	N/A	3	Dimensions	From data	Single
Carvalho 2018	ASD + HC	From data	Single	3	Dimensions	From data	Single
Chiappelli 2015a	SCZ + HC	Drawn	N/A	2	Volume	From data	Single
Chiappelli 2015b	SCZ + HC	Drawn	N/A	3	Dimensions	From data	Single
Chitty 2015	BD + HC	From data	Single	1	Dimensions	From data	Single

Chiu 2018	SCZ + HC	Drawn	N/A	3	N/A	From data	Single
Chouinard 2017	SCZ + HC	Drawn	N/A	1	Dimensions	From data	Single
Coughlin 2015	SCZ + HC	From data	Single	1	Dimensions	From data	Single
de Rezende 2018	PPD + HC	From data	Single	2	Dimensions	None	N/A
Drenthen 2016	High-functioning autism + HC	From data	Single	1	Dimensions	From data	Single
Dubin 2016	Depression (pre + post rTMS)	Drawn	N/A	1	Dimensions	From data	Single
Duffy 2015	“At risk” Depression + HC	From data	Single	1	Dimensions	From data	Single
Endres 2017	ASD + HC	From data	Single	1	Dimensions	From data	Single
Ernst 2017	MDD + HC	From data	Single	1	Dimensions	From data	Single
Evans 2018	MDD (baseline + post Ketamine treatment) + HC	From data	All subjects	2	Dimensions	From data	Single
Fleck 2017	BD-I (baseline + post Lithium treatment)	None	N/A	1	Dimensions	None	N/A
Ford 2017a	Social Disorganisation (ASD + SCZ trait phenotypes)	From data	Single	2	Dimensions	From data	Single
Ford 2017b	Social Disorganisation (ASD + SCZ trait phenotypes)	From data	Single	1	Dimensions	None	Group
Ford 2019	Social Disorganisation (ASD + SCZ trait phenotypes)	From data	Single	2	Dimensions	None	Group
Freed 2017	MDD + HC	Drawn	N/A	1	Dimensions	From data	Single

Gabbay 2017	MDD + HC	Drawn	N/A	1	Dimensions	From data	Single
Galinska-Skok 2018	SCZ + BD-I + HC	Drawn	N/A	2	Dimensions	From data	Single
Gallinat 2016	SCZ + HC	Drawn	N/A	1	Dimensions	From data	Single
Girgis 2019	SCZ + HC	Drawn	N/A	3	Dimensions	From data	Single
Godlewska 2018	BD (pre + post Lamotrigine)	From data	Single	2	Dimensions	None	N/A
Grent-'t-Jong 2018	FEP + SCZ+ HC	From data	Single	2	Dimensions	From data	Single
Haarman 2016	BD-I + HC	From data	Single	1	Dimensions	From data	Single
He 2018	High risk SCZ + HC	From data	Single	1	Dimensions	From data	Single
Henigsberg 2018	Recurrent depression (mono antidepressant treatment)	None	N/A	1	Dimensions	None	N/A
Horder 2018	ASD	Drawn	N/A	1	Dimensions	From data	Single
Huang 2017	First-episode SCZ	Drawn	N/A	1	Dimensions	From data	Single
Huber 2018	BD + HC	Drawn	N/A	1	Dimensions	From data	Single
Hugdahl 2015	SCZ + HC	Drawn	N/A	2	Dimensions	From data	Single
Inci Kenar 2017	ADHD (pre + post Methylphenidate)	None	N/A	1	N/A	None	N/A
Inci Kenar 2016	ADHD (pre + post Methylphenidate)	None	N/A	1	N/A	None	N/A
Ito 2017	ASD + HC	Drawn	N/A	1	Dimensions	From data	Single

Javitt 2018	HC (pre + post Ketamine)	Drawn	N/A	3	Dimensions	From data	Single
Jayaweera 2015	Life-time depression + HC	From data	Single	1	Dimensions	From data	Single
Jollant 2016	MDD + HC	From data	Group	1	Dimensions	From data	Single
Kim 2017	SCZ + BD + HC	Drawn	N/A	1	Dimensions	From data	Group
Kirtaş 2016	Chronic SCZ + FEP + HC	From data	Single	1	Dimensions	From data	Single
Klauser 2018	SCZ (placebo + N-acetylcysteine treatment)	None	N/A	1	Dimensions	None	N/A
Knudsen 2019	MDD (pre + post ECT)	From data	Single	3	Dimensions	From data	Single
Kubo 2017	BD + HC	From data	Single	3	Volume	From data	Single
Lee 2018	SCZ	None	N/A	1	Volume	None	N/A
Lewis 2016	Depression	Drawn	N/A	3	Dimensions	None	N/A
Libero 2015	ASD + HC	From data	Single	3	Dimensions	From data	Single
Lirng 2015	Migraine with MDD + migraine without MDD	Drawn	N/A	1	Dimensions	From data	Single
Liu 2015	SCZ + HC	Drawn	N/A	1	Dimensions	From data	Single
McQueen 2018	SCZ (placebo + N-acetylcysteine treatment)	Drawn	N/A	1	Dimensions	From data	Single
Milak 2016	MDD (pre + post Ketamine treatment)	Drawn	N/A	1	Dimensions	From data	Single
Mori 2015	ASD + HC	Drawn	N/A	1	Dimensions	From data	Single

Murrough 2018	BD (pre + post Minocycline treatment)	Drawn	N/A	2	Dimensions	From data	Single
Naaijen 2017	ADHD + HC	From data	Single	3	Volume	From data	Single
Naaijen 2018	ADHD + HC	From data	All subjects	3	Dimensions	From data	Single
Njau 2016	Depression + HC	Drawn	N/A	1	Dimensions	From data	Single
Ota 2015	SCZ + HC	None	N/A	1	Dimensions	None	N/A
Plitman 2018	SCZ + HC	From data	Single	3	Volume	None	N/A
Port 2017	ASD + HC	None	N/A	1	Dimensions	None	N/A
Posporelis 2018	SCZ + HC	From data	Single	3	Dimensions	From data	Single
Prisciandaro 2017	BD + alcohol dependence	From data	Single	3	Dimensions	From data	Single
Psomiades 2018	SCZ (AVH + non-AVH)	From data	N/A	1	Dimensions	From data	Single
Puts 2017	ASD + HC	Drawn	N/A	3	Dimensions	None	All subjects
Reid 2016	SCZ + HC	None	N/A	1	Dimensions	None	N/A
Rigucci 2018	Early psychosis (cannabis users) + HC	Drawn	N/A	1	Dimensions	From data	Single
Robertson 2016	ASD + HC	From data	Single	2	Dimensions	From data	All subjects
Rosa 2017	PPD + HC	From data	Single	3	Dimensions	From data	Single
Rowland 2016a	SCZ + HC	From data	Single	3	Dimensions	From data	Single
Rowland 2016b	SCZ + HC	From data	Single	3	Dimensions	From data	Single

Shirayama 2017	MDD + HC	From data	Single	1	Dimensions	None	N/A
Silveira 2016	BD + HC	Drawn	N/A	3	Dimensions	From data	Single
Singh 2018	SCZ + HC	From data	Single	1	Dimensions	From data	Single
Soeiro-de-Souza 2018	BD-I + HC	Drawn	N/A	3	Dimensions	From data	Single
Soeiro-de-Souza 2016	BD-I + HC	From data	Single	1	Dimensions	None	N/A
Solleveld 2017	ADHD (pre + post Methylphenidate)	From data	Single	3	Dimensions	From data	Single
Stivaros 2018	ASD with NF1 (Simvastatin + Placebo)	From data	Single	1	Dimensions	None	N/A
Strzelecki 2015	SCZ (antipsychotic + antidepressant treatment)	From data	Single	1	Dimensions	From data	Single
Thakkar 2017	SCZ + HC	Drawn	N/A	2	Dimensions	From data	Single
Ublinskii 2015	SCZ	Drawn	N/A	2	Dimensions	From data	Single
Unal 2016	ADHD (pre + post Methylphenidate)	None	N/A	1	N/A	None	N/A
Unal 2015	ADHD (pre + post Methylphenidate)	None	N/A	1	N/A	None	N/A
Urrila 2017	Depression + HC	From data	Single	1	Dimensions	From data	Single
Wang 2019	FEP + HC	Drawn	N/A	1	Dimensions	From data	Single
Wijtenburg 2017	SCZ + HC	From data	Single	1	Dimensions	From data	Single

Wise 2018	MDD + BD + HC	Drawn	N/A	3	Dimensions	None	N/A
Xia 2018	SCA (ECT + antipsychotic treatment)	From data	Single	1	Dimensions	From data	Single
Yang 2016	MDD + HC	Drawn	N/A	1	Volume	From data	Single
Yuksel 2015	BD-I + HC	Drawn	N/A	1	Dimensions	From data	Single
Zhang 2016	MDD + HC	Drawn	N/A	3	Dimensions	From data	Single
Zong 2015	FEP (pre +post Risperidone treatment) + HC	From data	Single	1	Dimensions	From data	Single

Supporting table 1: Studies included in the literature appraisal. Study references are given, along with the participant groups included in each. The type of voxel location information provided is then detailed. “From data” means that an actual MRS voxel from at least one participant is shown. “Drawn” means that a representation of the region has been made by the researcher rather than produced from data. Textual descriptions are rated into categories 1, 2, and 3, as detailed in the main text. The type of spectral images included are then listed. For both voxel location and spectra, where data is shown it is denoted whether this comes from a single participant, all participants irrespective of group membership, or with the participant groups separated.

AVH = Auditory verbal hallucinations
BD-I = Bipolar disorder I
BD-II = Bipolar disorder II
ECT = Electroconvulsive therapy
FEP = First-episode psychosis
NF1 = Neurofibromatosis 1
PPD = Postpartum Depression

References

Aoki, Y., Watanabe, T., Abe, O., Kuwabara, H., Yahata, N., Takano, Y., Iwashiro, N., Natsubori, T., Takao, H., Kawakubo, Y., Kasai, K., Yamasue, H., 2015. Oxytocin's neurochemical effects in the medial prefrontal cortex underlie recovery of task-specific brain activity in autism: a randomized controlled trial. Mol. Psychiatry 20, 447–453. https://doi.org/10.1038/mp.2014.74

Atagun, M.I., Sikoglu, E.M., Can, S.S., Karakas-Ugurlu, G., Ulusoy-Kaymak, S., Caykoylu, A., Algin, O., Phillips, M.L., Moore, C.M., Ongur, D., 2015. Investigation of Heschl's gyrus and planum temporale in patients with schizophrenia and bipolar disorder: a proton magnetic resonance spectroscopy study. Schizophr. Res. 161, 202–209. https://doi.org/10.1016/j.schres.2014.11.012

Atagun, M.I., Sikoglu, E.M., Can, S.S., Ugurlu, G.K., Kaymak, S.U., Caykoylu, A., Algin, O., Phillips, M.L., Moore, C.M., Ongur, D., 2018. Neurochemical differences between bipolar disorder type I and II in superior temporal cortices: A proton magnetic resonance spectroscopy study. J. Affect. Disord. 235, 15–19. https://doi.org/10.1016/j.jad.2018.04.010

Atagun, M.I., Sikoglu, E.M., Soykan, C., Serdar Suleyman, C., Ulusoy-Kaymak, S., Caykoylu, A., Algin, O., Phillips, M.L., Ongur, D., Moore, C.M., 2017. Perisylvian GABA levels in schizophrenia and bipolar disorder. Neurosci. Lett. 637, 70–74. https://doi.org/10.1016/j.neulet.2016.11.051

Bae, S., Han, D.H., Kim, S.M., Shi, X., Renshaw, P.F., 2016. Neurochemical correlates of internet game play in adolescents with attention deficit hyperactivity disorder: A proton magnetic resonance spectroscopy (MRS) study. Psychiatry Res. Neuroimaging 254, 10–17. https://doi.org/10.1016/j.pscychresns.2016.05.006

Bauer, J., Werner, A., Kohl, W., Kugel, H., Shushakova, A., Pedersen, A., Ohrmann, P., 2016. Hyperactivity and impulsivity in adult attention-deficit/hyperactivity disorder is related to glutamatergic dysfunction in the anterior cingulate cortex. World J. Biol. Psychiatry Off. J. World Fed. Soc. Biol. Psychiatry 19, 538–546. https://doi.org/10.1080/15622975.2016.1262060

Bollmann, S., Ghisleni, C., Poil, S.-S., Martin, E., Ball, J., Eich-Hochli, D., Edden, R.A.E., Klaver, P., Michels, L., Brandeis, D., O'Gorman, R.L., 2015. Developmental changes in gamma-aminobutyric acid levels in attention-deficit/hyperactivity disorder. Transl. Psychiatry 5, e589. https://doi.org/10.1038/tp.2015.79

Bond, D.J., Silveira, L.E. da, MacMillan, E.L., Torres, I.J., Lang, D.J., Su, W., Honer, W.G., Lam, R.W., Yatham, L.N., 2016. Relationship between body mass index and hippocampal glutamate/glutamine in bipolar disorder. Br. J. Psychiatry 208, 146–152. https://doi.org/10.1192/bjp.bp.115.163360

Bossong, M.G., Antoniades, M., Azis, M., Samson, C., Quinn, B., Bonoldi, I., Modinos, G., Perez, J., Howes, O.D., Stone, J.M., Allen, P., McGuire, P., 2019. Association of Hippocampal Glutamate Levels With Adverse Outcomes in Individuals at Clinical High Risk for Psychosis. JAMA Psychiatry 76, 199–207. https://doi.org/10.1001/jamapsychiatry.2018.3252

Brennan, B.P., Admon, R., Perriello, C., LaFlamme, E.M., Athey, A.J., Pizzagalli, D.A., Hudson, J.I., Pope, H.G., Jensen, J.E., 2017. Acute change in anterior cingulate cortex GABA, but not glutamine/glutamate, mediates antidepressant response to citalopram. Psychiatry Res. Neuroimaging 269, 9–16. https://doi.org/10.1016/j.pscychresns.2017.08.009

Bustillo, J.R., Rediske, N., Jones, T., Rowland, L.M., Abbott, C., Wijtenburg, S.A., 2016. Reproducibility of phase rotation stimulated echo acquisition mode at 3T in schizophrenia: Emphasis on glutamine. Magn. Reson. Med. 75, 498–502. https://doi.org/10.1002/mrm.25638

Carvalho Pereira, A., Violante, I.R., Mouga, S., Oliveira, G., Castelo-Branco, M., 2018. Medial Frontal Lobe Neurochemistry in Autism Spectrum Disorder is Marked by Reduced N-Acetylaspartate and Unchanged Gamma-Aminobutyric Acid and Glutamate + Glutamine Levels. J. Autism Dev. Disord. 48, 1467–1482. https://doi.org/10.1007/s10803-017-3406-8

Chiappelli, J., Hong, L.E., Wijtenburg, S.A., Du, X., Gaston, F., Kochunov, P., Rowland, L.M., 2015a. Alterations in frontal white matter neurochemistry and microstructure in schizophrenia: implications for neuroinflammation. Transl. Psychiatry 5, e548–e548. https://doi.org/10.1038/tp.2015.43

Chiappelli, J., Rowland, L.M., Wijtenburg, S.A., Muellerklein, F., Tagamets, M., McMahon, R.P., Gaston, F., Kochunov, P., Hong, L.E., 2015b. Evaluation of Myo-Inositol as a Potential Biomarker for Depression in Schizophrenia. Neuropsychopharmacology 40, 2157–2164. https://doi.org/10.1038/npp.2015.57

Chitty, K.M., Lagopoulos, J., Hickie, I.B., Hermens, D.F., 2015. Investigating the role of glutathione in mismatch negativity: An insight into NMDA receptor disturbances in bipolar disorder. Clin. Neurophysiol. 126, 1178–1184. https://doi.org/10.1016/j.clinph.2014.09.025

Chiu, P.W., Lui, S.S.Y., Hung, K.S.Y., Chan, R.C.K., Chan, Q., Sham, P.C., Cheung, E.F.C., Mak, H.K.F., 2018. In vivo gamma-aminobutyric acid and glutamate levels in people with first-episode schizophrenia: A proton magnetic resonance spectroscopy study. Schizophr. Res. 193, 295–303. https://doi.org/10.1016/j.schres.2017.07.021

Chouinard, V.-A., Kim, S.-Y., Valeri, L., Yuksel, C., Ryan, K.P., Chouinard, G., Cohen, B.M., Du, F., Ongur, D., 2017. Brain bioenergetics and redox state measured by (31)P magnetic resonance spectroscopy in unaffected siblings of patients with psychotic disorders. Schizophr. Res. 187, 11–16. https://doi.org/10.1016/j.schres.2017.02.024

Coughlin, J.M., Tanaka, T., Marsman, A., Wang, H., Bonekamp, S., Kim, P.K., Higgs, C., Varvaris, M., Edden, R. a. E., Pomper, M., Schretlen, D., Barker, P.B., Sawa, A., 2015. Decoupling of N-acetyl-aspartate and glutamate within the dorsolateral prefrontal cortex in schizophrenia. Curr. Mol. Med. 15, 176–183. https://doi.org/10.2174/1566524015666150303104811

de Rezende, M.G., Rosa, C.E., Garcia-Leal, C., de Figueiredo, F.P., Cavalli, R. de C., Bettiol, H., Salmon, C.E.G., Barbieri, M.A., de Castro, M., Carlos dos Santos, A., Del-Ben, C.M., 2018. Correlations between changes in the hypothalamic-pituitary-adrenal axis and neurochemistry of the anterior cingulate gyrus in postpartum depression. J. Affect. Disord. 239, 274–281. https://doi.org/10.1016/j.jad.2018.07.028

Drenthen, G.S., Barendse, E.M., Aldenkamp, A.P., van Veenendaal, T.M., Puts, N.A.J., Edden, R.A.E., Zinger, S., Thoonen, G., Hendriks, M.P.H., Kessels, R.P.C., Jansen, J.F.A., 2016. Altered neurotransmitter metabolism in adolescents with high-functioning autism. Psychiatry Res. Neuroimaging 256, 44–49. https://doi.org/10.1016/j.pscychresns.2016.09.007

Dubin, M.J., Mao, X., Banerjee, S., Goodman, Z., Lapidus, K.A.B., Kang, G., Liston, C., Shungu, D.C., 2016. Elevated prefrontal cortex GABA in patients with major depressive disorder after TMS treatment measured with proton magnetic resonance spectroscopy. J. Psychiatry Neurosci. JPN 41, E37-45. https://doi.org/10.1503/jpn.150223

Duffy, S.L., Lagopoulos, J., Cockayne, N., Hermens, D.F., Hickie, I.B., Naismith, S.L., 2015. Oxidative stress and depressive symptoms in older adults: A magnetic resonance spectroscopy study. J. Affect. Disord. 180, 29–35. https://doi.org/10.1016/j.jad.2015.03.007

Endres, D., Tebartz van Elst, L., Meyer, S.A., Feige, B., Nickel, K., Bubl, A., Riedel, A., Ebert, D., Lange, T., Glauche, V., Biscaldi, M.,

Philipsen, A., Maier, S.J., Perlov, E., 2017. Glutathione metabolism in the prefrontal brain of adults with high-functioning autism spectrum disorder: an MRS study. Mol. Autism 8. https://doi.org/10.1186/s13229-017-0122-3

Ernst, J., Hock, A., Henning, A., Seifritz, E., Boeker, H., Grimm, S., 2017. Increased pregenual anterior cingulate glucose and lactate concentrations in major depressive disorder. Mol. Psychiatry 22, 113–119. https://doi.org/10.1038/mp.2016.73

Evans, J.W., Lally, N., An, L., Li, N., Nugent, A.C., Banerjee, D., Snider, S.L., Shen, J., Roiser, J.P., Zarate, C.A.J., 2018. 7T (1)H-MRS in major depressive disorder: a Ketamine Treatment Study. Neuropsychopharmacol. Off. Publ. Am. Coll. Neuropsychopharmacol. 43, 1908–1914. https://doi.org/10.1038/s41386-018-0057-1

Fleck, D.E., Ernest, N., Adler, C.M., Cohen, K., Eliassen, J.C., Norris, M., Komoroski, R.A., Chu, W.-J., Welge, J.A., Blom, T.J., DelBello, M.P., Strakowski, S.M., 2017. Prediction of lithium response in first-episode mania using the LITHium Intelligent Agent (LITHIA): Pilot data and proof-of-concept. Bipolar Disord. 19, 259–272. https://doi.org/10.1111/bdi.12507

Ford, T.C., Abu-Akel, A., Crewther, D.P., 2019. The association of excitation and inhibition signaling with the relative symptom expression of autism and psychosis-proneness: Implications for psychopharmacology. Prog. Neuropsychopharmacol. Biol. Psychiatry 88, 235–242. https://doi.org/10.1016/j.pnpbp.2018.07.024

Ford, T.C., Nibbs, R., Crewther, D.P., 2017a. Increased glutamate/GABA+ ratio in a shared autistic and schizotypal trait phenotype termed Social Disorganisation. NeuroImage Clin. 16, 125–131. https://doi.org/10.1016/j.nicl.2017.07.009

Ford, T.C., Nibbs, R., Crewther, D.P., 2017b. Glutamate/GABA+ ratio is associated with the psychosocial domain of autistic and schizotypal traits. PloS One 12, e0181961. https://doi.org/10.1371/journal.pone.0181961

Freed, R.D., Hollenhorst, C.N., Weiduschat, N., Mao, X., Kang, G., Shungu, D.C., Gabbay, V., 2017. A pilot study of cortical glutathione in youth with depression. Psychiatry Res. Neuroimaging 270, 54–60. https://doi.org/10.1016/j.pscychresns.2017.10.001

Gabbay, V., Bradley, K.A., Mao, X., Ostrover, R., Kang, G., Shungu, D.C., 2017. Anterior cingulate cortex γ-aminobutyric acid deficits in youth with depression. Transl. Psychiatry 7, e1216–e1216. https://doi.org/10.1038/tp.2017.187

Galinska-Skok, B., Malus, A., Konarzewska, B., Rogowska-Zach, A., Milewski, R., Tarasow, E., Szulc, A., Waszkiewicz, N., 2018. Choline Compounds of the Frontal Lobe and Temporal Glutamatergic System in Bipolar and Schizophrenia Proton Magnetic Resonance Spectroscopy Study. Dis. Markers 2018, 3654894. https://doi.org/10.1155/2018/3654894

Gallinat, J., McMahon, K., Kühn, S., Schubert, F., Schaefer, M., 2016. Cross-sectional Study of Glutamate in the Anterior Cingulate and Hippocampus in Schizophrenia. Schizophr. Bull. 42, 425–433. https://doi.org/10.1093/schbul/sbv124

Girgis, R.R., Baker, S., Mao, X., Gil, R., Javitt, D.C., Kantrowitz, J.T., Gu, M., Spielman, D.M., Ojeil, N., Xu, X., Abi-Dargham, A., Shungu, D.C., Kegeles, L.S., 2019. Effects of acute N-acetylcysteine challenge on cortical glutathione and glutamate in schizophrenia: A pilot in vivo proton magnetic resonance spectroscopy study. Psychiatry Res. 275, 78–85. https://doi.org/10.1016/j.psychres.2019.03.018

Godlewska, B.R., Masaki, C., Sharpley, A.L., Cowen, P.J., Emir, U.E., 2018. Brain glutamate in medication-free depressed patients: a proton MRS study at 7 Tesla. Psychol. Med. 48, 1731–1737. https://doi.org/10.1017/S0033291717003373

Grent-'t-Jong, T., Gross, J., Goense, J., Wibral, M., Gajwani, R., Gumley, A.I., Lawrie, S.M., Schwannauer, M., Schultze-Lutter, F., Navarro Schröder, T., Koethe, D., Leweke, F.M., Singer, W., Uhlhaas, P.J., 2018. Resting-state gamma-band power alterations in schizophrenia

reveal E/I-balance abnormalities across illness-stages. eLife 7, e37799. https://doi.org/10.7554/eLife.37799
Haarman, B.C.M. “Benno,” Burger, H., Doorduin, J., Renken, R.J., Sibeijn-Kuiper, A.J., Marsman, J.-B.C., de Vries, E.F.J., de Groot, J.C., Drexhage, H.A., Mendes, R., Nolen, W.A., Riemersma-Van der Lek, R.F., 2016. Volume, metabolites and neuroinflammation of the hippocampus in bipolar disorder - A combined magnetic resonance imaging and positron emission tomography study. Brain. Behav. Immun. 56, 21–33. https://doi.org/10.1016/j.bbi.2015.09.004
He, Y., Kosciolek, T., Tang, J., Zhou, Y., Li, Z., Ma, X., Zhu, Q., Yuan, N., Yuan, L., Li, C., Jin, K., Knight, R., Tsuang, M.T., Chen, X., 2018. Gut microbiome and magnetic resonance spectroscopy study of subjects at ultra-high risk for psychosis may support the membrane hypothesis. Eur. Psychiatry 53, 37–45. https://doi.org/10.1016/j.eurpsy.2018.05.011
Henigsberg, N., Savić, A., Radoš, Marko, Šarac, H., Radoš, Milan, Ozretić, D., Bajs Janović, M., Erdeljić Turk, V., Šečić, A., Kalember, P., Hrabač, P., 2018. Choline and N-acetyl aspartate levels in the dorsolateral prefrontal cortex at the beginning of the recovery phase as markers of increased risk for depressive episode recurrence under different duration of maintenance therapy and after it: a retrospective cohort study. Croat. Med. J. 59, 244–252. https://doi.org/10.3325/cmj.2018.59.244
Horder, J., Petrinovic, M.M., Mendez, M.A., Bruns, A., Takumi, T., Spooren, W., Barker, G.J., Kunnecke, B., Murphy, D.G., 2018. Glutamate and GABA in autism spectrum disorder-a translational magnetic resonance spectroscopy study in man and rodent models. Transl. Psychiatry 8, 106. https://doi.org/10.1038/s41398-018-0155-1
Huang, M.-L., Khoh, T.-T., Lu, S.-J., Pan, F., Chen, J.-K., Hu, J.-B., Hu, S.-H., Xu, W.-J., Zhou, W.-H., Wei, N., Qi, H.-L., Shang, D.-S., Xu, Y., 2017. Relationships between dorsolateral prefrontal cortex metabolic change and cognitive impairment in first-episode neuroleptic-naive schizophrenia patients. Medicine (Baltimore) 96, e7228. https://doi.org/10.1097/MD.0000000000007228
Huber, R.S., Kondo, D.G., Shi, X.-F., Prescot, A.P., Clark, E., Renshaw, P.F., Yurgelun-Todd, D.A., 2018. Relationship of executive functioning deficits to N-acetyl aspartate (NAA) and gamma-aminobutyric acid (GABA) in youth with bipolar disorder. J. Affect. Disord. 225, 71–78. https://doi.org/10.1016/j.jad.2017.07.052
Hugdahl, K., Craven, A.R., Nygård, M., Løberg, E.-M., Berle, J.Ø., Johnsen, E., Kroken, R., Specht, K., Andreassen, O.A., Ersland, L., 2015. Glutamate as a mediating transmitter for auditory hallucinations in schizophrenia: A 1H MRS study. Schizophr. Res. 161, 252–260. https://doi.org/10.1016/j.schres.2014.11.015
Inci Kenar, A.N., Ünal, G.A., Güler, H., Albuz, B., Kıroğlu, Y., Erdal, M.E., Herken, H., 2016. Relationship between the DAT1 gene and the effects of methylphenidate administration in adult attention deficit hyperactivity disorder: a magnetic resonance spectroscopy study. Eur. Rev. Med. Pharmacol. Sci. 20, 1373–1378.
Inci Kenar, A.N., Unal, G.A., Kiroglu, Y., Herken, H., 2017. Effects of methylphenidate treatment on the cerebellum in adult attention-deficit hyperactivity disorder: a magnetic resonance spectroscopy study. Eur. Rev. Med. Pharmacol. Sci. 21, 383–388.
Ito, H., Mori, K., Harada, M., Hisaoka, S., Toda, Y., Mori, T., Goji, A., Abe, Y., Miyazaki, M., Kagami, S., 2017. A Proton Magnetic Resonance Spectroscopic Study in Autism Spectrum Disorder Using a 3-Tesla Clinical Magnetic Resonance Imaging (MRI) System: The Anterior Cingulate Cortex and the Left Cerebellum. J. Child Neurol. 32, 731–739. https://doi.org/10.1177/0883073817702981
Javitt, D.C., Carter, C.S., Krystal, J.H., Kantrowitz, J.T., Girgis, R.R., Kegeles, L.S., Ragland, J.D., Maddock, R.J., Lesh, T.A., Tanase, C.,

Corlett, P.R., Rothman, D.L., Mason, G., Qiu, M., Robinson, J., Potter, W.Z., Carlson, M., Wall, M.M., Choo, T.-H., Grinband, J., Lieberman, J.A., 2018. Utility of Imaging-Based Biomarkers for Glutamate-Targeted Drug Development in Psychotic Disorders: A Randomized Clinical Trial. JAMA Psychiatry 75, 11–19. https://doi.org/10.1001/jamapsychiatry.2017.3572

Jayaweera, H.K., Lagopoulos, J., Duffy, S.L., Lewis, S.J.G., Hermens, D.F., Norrie, L., Hickie, I.B., Naismith, S.L., 2015. Spectroscopic markers of memory impairment, symptom severity and age of onset in older people with lifetime depression: Discrete roles of N-acetyl aspartate and glutamate. J. Affect. Disord. 183, 31–38. https://doi.org/10.1016/j.jad.2015.04.023

Jollant, F., Richard-Devantoy, S., Ding, Y., Turecki, G., Bechara, A., Near, J., 2016. Prefrontal inositol levels and implicit decision-making in healthy individuals and depressed patients. Eur. Neuropsychopharmacol. 26, 1255–1263. https://doi.org/10.1016/j.euroneuro.2016.06.005

Kim, S.-Y., Cohen, B.M., Chen, X., Lukas, S.E., Shinn, A.K., Yuksel, A.C., Li, T., Du, F., Ongur, D., 2017. Redox Dysregulation in Schizophrenia Revealed by in vivo NAD+/NADH Measurement. Schizophr. Bull. 43, 197–204. https://doi.org/10.1093/schbul/sbw129

Kirtaş, D., Karadağ, R.F., Balci Şengül, M.C., Kiroğlu, Y., 2016. 1H-magnetic resonance spectroscopy in first episode and chronic schizophrenia patients. Turk. J. Med. Sci. 46, 862–871. https://doi.org/10.3906/sag-1502-9

Klauser, P., Xin, L., Fournier, M., Griffa, A., Cleusix, M., Jenni, R., Cuenod, M., Gruetter, R., Hagmann, P., Conus, P., Baumann, P.S., Do, K.Q., 2018. N -acetylcysteine add-on treatment leads to an improvement of fornix white matter integrity in early psychosis: a double-blind randomized placebo-controlled trial. Transl. Psychiatry 8, 1–8. https://doi.org/10.1038/s41398-018-0266-8

Knudsen, M.K., Near, J., Blicher, A.B., Videbech, P., Blicher, J.U., 2019. Magnetic resonance (MR) spectroscopic measurement of γ-aminobutyric acid (GABA) in major depression before and after electroconvulsive therapy. Acta Neuropsychiatr. 31, 17–26. https://doi.org/10.1017/neu.2018.22

Kubo, H., Nakataki, M., Sumitani, S., Iga, J., Numata, S., Kameoka, N., Watanabe, S., Umehara, H., Kinoshita, M., Inoshita, M., Tamaru, M., Ohta, M., Nakayama-Yamauchi, C., Funakoshi, Y., Harada, M., Ohmori, T., 2017. 1H-magnetic resonance spectroscopy study of glutamate-related abnormality in bipolar disorder. J. Affect. Disord. 208, 139–144. https://doi.org/10.1016/j.jad.2016.08.046

Lee, J., Yoon, Y.B., Wijtenburg, S.A., Rowland, L.M., Chen, H., Gaston, F.E., Song, I.C., Cho, K.I.K., Kim, M., Lee, T.Y., Kwon, J.S., 2018. Lower glutamate level in temporo-parietal junction may predict a better response to tDCS in schizophrenia. Schizophr. Res. 201, 422–423. https://doi.org/10.1016/j.schres.2018.05.032

Lewis, C.P., Port, J.D., Frye, M.A., Vande Voort, J.L., Ameis, S.H., Husain, M.M., Daskalakis, Z.J., Croarkin, P.E., 2016. An Exploratory Study of Spectroscopic Glutamatergic Correlates of Cortical Excitability in Depressed Adolescents. Front. Neural Circuits 10, 98. https://doi.org/10.3389/fncir.2016.00098

Libero, L.E., Reid, M.A., White, D.M., Salibi, N., Lahti, A.C., Kana, R.K., 2015. Biochemistry of the cingulate cortex in autism: An MR spectroscopy study. Autism Res. Off. J. Int. Soc. Autism Res. 9, 643–657. https://doi.org/10.1002/aur.1562

Lirng, J.-F., Chen, H.-C., Fuh, J.-L., Tsai, C.-F., Liang, J.-F., Wang, S.-J., 2015. Increased myo-inositol level in dorsolateral prefrontal cortex in migraine patients with major depression. Cephalalgia 35, 702–709. https://doi.org/10.1177/0333102414557048

Liu, W., Yu, H., Jiang, B., Pan, B., Yu, S., Li, H., Zheng, L., 2015. The predictive value of baseline NAA/Cr for treatment response of

first-episode schizophrenia: A 1H MRS study. Neurosci. Lett. 600, 199–205. https://doi.org/10.1016/j.neulet.2015.06.029
McQueen, G., Lally, J., Collier, T., Zelaya, F., Lythgoe, D.J., Barker, G.J., Stone, J.M., McGuire, P., MacCabe, J.H., Egerton, A., 2018. Effects of N-acetylcysteine on brain glutamate levels and resting perfusion in schizophrenia. Psychopharmacology (Berl.) 235, 3045–3054. https://doi.org/10.1007/s00213-018-4997-2
Milak, M.S., Proper, C.J., Mulhern, S.T., Parter, A.L., Kegeles, L.S., Ogden, R.T., Mao, X., Rodriguez, C.I., Oquendo, M.A., Suckow, R.F., Cooper, T.B., Keilp, J.G., Shungu, D.C., Mann, J.J., 2016. A pilot in vivo proton magnetic resonance spectroscopy study of amino acid neurotransmitter response to ketamine treatment of major depressive disorder. Mol. Psychiatry 21, 320–327. https://doi.org/10.1038/mp.2015.83
Mori, Kenji, Toda, Y., Ito, H., Mori, T., Mori, Keiko, Goji, A., Hashimoto, H., Tani, H., Miyazaki, M., Harada, M., Kagami, S., 2015. Neuroimaging in autism spectrum disorders: 1H-MRS and NIRS study. J. Med. Investig. JMI 62, 29–36. https://doi.org/10.2152/jmi.62.29
Murrough, J.W., Huryk, K.M., Mao, X., Iacoviello, B., Collins, K., Nierenberg, A.A., Kang, G., Shungu, D.C., Iosifescu, D.V., 2018. A pilot study of minocycline for the treatment of bipolar depression: Effects on cortical glutathione and oxidative stress in vivo. J. Affect. Disord. 230, 56–64. https://doi.org/10.1016/j.jad.2017.12.067
Naaijen, J., Forde, N.J., Lythgoe, D.J., Akkermans, S.E.A., Openneer, T.J.C., Dietrich, A., Zwiers, M.P., Hoekstra, P.J., Buitelaar, J.K., 2017. Fronto-striatal glutamate in children with Tourette's disorder and attention-deficit/hyperactivity disorder. NeuroImage Clin. 13, 16–23. https://doi.org/10.1016/j.nicl.2016.11.013
Naaijen, J., Lythgoe, D.J., Zwiers, M.P., Hartman, C.A., Hoekstra, P.J., Buitelaar, J.K., Aarts, E., 2018. Anterior cingulate cortex glutamate and its association with striatal functioning during cognitive control. Eur. Neuropsychopharmacol. J. Eur. Coll. Neuropsychopharmacol. 28, 381–391. https://doi.org/10.1016/j.euroneuro.2018.01.002
Njau, S., Joshi, S.H., Leaver, A.M., Vasavada, M., Van Fleet, J., Espinoza, R., Narr, K.L., 2016. Variations in myo-inositol in fronto-limbic regions and clinical response to electroconvulsive therapy in major depression. J. Psychiatr. Res. 80, 45–51. https://doi.org/10.1016/j.jpsychires.2016.05.012
Ota, M., Wakabayashi, C., Sato, N., Hori, H., Hattori, K., Teraishi, T., Ozawa, H., Okubo, T., Kunugi, H., 2015. Effect of L-theanine on glutamatergic function in patients with schizophrenia. Acta Neuropsychiatr. 27, 291–296. https://doi.org/10.1017/neu.2015.22
Plitman, E., Chavez, S., Nakajima, S., Iwata, Y., Chung, J.K., Caravaggio, F., Kim, J., Alshehri, Y., Chakravarty, M.M., De Luca, V., Remington, G., Gerretsen, P., Graff-Guerrero, A., 2018. Striatal neurometabolite levels in patients with schizophrenia undergoing long-term antipsychotic treatment: A proton magnetic resonance spectroscopy and reliability study. Psychiatry Res. Neuroimaging 273, 16–24. https://doi.org/10.1016/j.pscychresns.2018.01.004
Port, R.G., Gaetz, W., Bloy, L., Wang, D.-J., Blaskey, L., Kuschner, E.S., Levy, S.E., Brodkin, E.S., Roberts, T.P.L., 2017. Exploring the relationship between cortical GABA concentrations, auditory gamma-band responses and development in ASD: Evidence for an altered maturational trajectory in ASD. Autism Res. 10, 593–607. https://doi.org/10.1002/aur.1686
Posporelis, S., Coughlin, J.M., Marsman, A., Pradhan, S., Tanaka, T., Wang, H., Varvaris, M., Ward, R., Higgs, C., Edwards, J.A., Ford, C.N., Kim, P.K., Lloyd, A.M., Edden, R.A.E., Schretlen, D.J., Cascella, N.G., Barker, P.B., Sawa, A., 2018. Decoupling of Brain Temperature

and Glutamate in Recent Onset of Schizophrenia: A 7T Proton Magnetic Resonance Spectroscopy Study. Biol. Psychiatry Cogn. Neurosci. Neuroimaging 3, 248–254. https://doi.org/10.1016/j.bpsc.2017.04.003

Prisciandaro, J.J., Tolliver, B.K., Prescot, A.P., Brenner, H.M., Renshaw, P.F., Brown, T.R., Anton, R.F., 2017. Unique prefrontal GABA and glutamate disturbances in co-occurring bipolar disorder and alcohol dependence. Transl. Psychiatry 7, e1163. https://doi.org/10.1038/tp.2017.141

Psomiades, M., Mondino, M., Fonteneau, C., Bation, R., Haesebaert, F., Suaud-Chagny, M.-F., Brunelin, J., 2018. N-Acetyl-Aspartate in the dorsolateral prefrontal cortex in men with schizophrenia and auditory verbal hallucinations: A 1.5 T Magnetic Resonance Spectroscopy Study. Sci. Rep. 8, 4133. https://doi.org/10.1038/s41598-018-22597-y

Puts, N.A.J., Wodka, E.L., Harris, A.D., Crocetti, D., Tommerdahl, M., Mostofsky, S.H., Edden, R.A.E., 2017. Reduced GABA and altered somatosensory function in children with autism spectrum disorder. Autism Res. Off. J. Int. Soc. Autism Res. 10, 608–619. https://doi.org/10.1002/aur.1691

Reid, M.A., White, D.M., Kraguljac, N.V., Lahti, A.C., 2016. A combined diffusion tensor imaging and magnetic resonance spectroscopy study of patients with schizophrenia. Schizophr. Res. 170, 341–350. https://doi.org/10.1016/j.schres.2015.12.003

Rigucci, S., Xin, L., Klauser, P., Baumann, P.S., Alameda, L., Cleusix, M., Jenni, R., Ferrari, C., Pompili, M., Gruetter, R., Do, K.Q., Conus, P., 2018. Cannabis use in early psychosis is associated with reduced glutamate levels in the prefrontal cortex. Psychopharmacology (Berl.) 235, 13–22. https://doi.org/10.1007/s00213-017-4745-z

Robertson, C.E., Ratai, E.-M., Kanwisher, N., 2016. Reduced GABAergic Action in the Autistic Brain. Curr. Biol. CB 26, 80–85. https://doi.org/10.1016/j.cub.2015.11.019

Rosa, C.E., Soares, J.C., Figueiredo, F.P., Cavalli, R.C., Barbieri, M.A., Schaufelberger, M.S., Salmon, C.E.G., Del-Ben, C.M., Santos, A.C., 2017. Glutamatergic and neural dysfunction in postpartum depression using magnetic resonance spectroscopy. Psychiatry Res. Neuroimaging 265, 18–25. https://doi.org/10.1016/j.pscychresns.2017.04.008

Rowland, L.M., Krause, B.W., Wijtenburg, S.A., McMahon, R.P., Chiappelli, J., Nugent, K.L., Nisonger, S.J., Korenic, S.A., Kochunov, P., Hong, L.E., 2016a. Medial frontal GABA is lower in older schizophrenia: a MEGA-PRESS with macromolecule suppression study. Mol. Psychiatry 21, 198–204. https://doi.org/10.1038/mp.2015.34

Rowland, L.M., Pradhan, S., Korenic, S., Wijtenburg, S.A., Hong, L.E., Edden, R.A., Barker, P.B., 2016b. Elevated brain lactate in schizophrenia: a 7 T magnetic resonance spectroscopy study. Transl. Psychiatry 6, e967–e967. https://doi.org/10.1038/tp.2016.239

Shirayama, Y., Takahashi, M., Osone, F., Hara, A., Okubo, T., 2017. Myo-inositol, Glutamate, and Glutamine in the Prefrontal Cortex, Hippocampus, and Amygdala in Major Depression. Biol. Psychiatry Cogn. Neurosci. Neuroimaging 2, 196–204. https://doi.org/10.1016/j.bpsc.2016.11.006

Silveira, L.E., Bond, D.J., MacMillan, E.L., Kozicky, J.-M., Muralidharan, K., Bucker, J., Rosa, A.R., Kapczinski, F., Yatham, L.N., 2016. Hippocampal neurochemical markers in bipolar disorder patients following the first-manic episode: A prospective 12-month proton magnetic resonance spectroscopy study. Aust. N. Z. J. Psychiatry 51, 65–74. https://doi.org/10.1177/0004867415623859

Singh, S., Khushu, S., Kumar, P., Goyal, S., Bhatia, T., Deshpande, S.N., 2018. Evidence for regional hippocampal damage in patients with

schizophrenia. Neuroradiology 60, 199–205. https://doi.org/10.1007/s00234-017-1954-4
Soeiro-de-Souza, M.G., Otaduy, M.C.G., Machado-Vieira, R., Moreno, R.A., Nery, F.G., Leite, C., Lafer, B., 2018. Anterior Cingulate Cortex Glutamatergic Metabolites and Mood Stabilizers in Euthymic Bipolar I Disorder Patients: A Proton Magnetic Resonance Spectroscopy Study. Biol. Psychiatry Cogn. Neurosci. Neuroimaging 3, 985–991. https://doi.org/10.1016/j.bpsc.2018.02.007
Soeiro-de-Souza, M.G., Pastorello, B.F., Leite, C. da C., Henning, A., Moreno, R.A., Garcia Otaduy, M.C., 2016. Dorsal Anterior Cingulate Lactate and Glutathione Levels in Euthymic Bipolar I Disorder: 1H-MRS Study. Int. J. Neuropsychopharmacol. 19. https://doi.org/10.1093/ijnp/pyw032
Solleveld, M.M., Schrantee, A., Puts, N.A.J., Reneman, L., Lucassen, P.J., 2017. Age-dependent, lasting effects of methylphenidate on the GABAergic system of ADHD patients. NeuroImage Clin. 15, 812–818. https://doi.org/10.1016/j.nicl.2017.06.003
Stivaros, S., Garg, S., Tziraki, M., Cai, Y., Thomas, O., Mellor, J., Morris, A.A., Jim, C., Szumanska-Ryt, K., Parkes, L.M., Haroon, H.A., Montaldi, D., Webb, N., Keane, J., Castellanos, F.X., Silva, A.J., Huson, S., Williams, S., Gareth Evans, D., Emsley, R., Green, J., 2018. Randomised controlled trial of simvastatin treatment for autism in young children with neurofibromatosis type 1 (SANTA). Mol. Autism 9, 12. https://doi.org/10.1186/s13229-018-0190-z
Strzelecki, D., Grzelak, P., Podgórski, M., Kałużyńska, O., Stefańczyk, L., Kotlicka-Antczak, M., Gmitrowicz, A., 2015. Comparison of Metabolite Concentrations in the Left Dorsolateral Prefrontal Cortex, the Left Frontal White Matter, and the Left Hippocampus in Patients in Stable Schizophrenia Treated with Antipsychotics with or without Antidepressants. 1H-NMR Spectroscopy Study. Int. J. Mol. Sci. 16, 24387–24402. https://doi.org/10.3390/ijms161024387
Thakkar, K.N., Rosler, L., Wijnen, J.P., Boer, V.O., Klomp, D.W.J., Cahn, W., Kahn, R.S., Neggers, S.F.W., 2017. 7T Proton Magnetic Resonance Spectroscopy of Gamma-Aminobutyric Acid, Glutamate, and Glutamine Reveals Altered Concentrations in Patients With Schizophrenia and Healthy Siblings. Biol. Psychiatry 81, 525–535. https://doi.org/10.1016/j.biopsych.2016.04.007
Ublinskii, M.V., Semenova, N.A., Lukovkina, O.V., Sidorin, S.V., Lebedeva, I.S., Akhadov, T.A., 2015. Diffusion in the Corpus Callosum in Patients with Early Schizophrenia. Bull. Exp. Biol. Med. 158, 611–613. https://doi.org/10.1007/s10517-015-2818-8
Unal, G.A., Inci Kenar, A.N., Tepeli, E., Kiroglu, Y., Herken, H., 2016. Relationship between the SNAP-25 gene and the effects of methylphenidate on the anterior cingulate cortex of patients with adult attention deficit hyperactivity disorder: a magnetic resonance spectroscopy study. Eur. Rev. Med. Pharmacol. Sci. 20, 2443–2449.
Unal, G.A., Kenar, A.N.I., Herken, H., Kiroglu, Y., 2015. Association of adult attention deficit hyperactivity disorder subtypes and response to methylphenidate HCL treatment: A magnetic resonance spectroscopy study. Neurosci. Lett. 604, 188–192. https://doi.org/10.1016/j.neulet.2015.08.006
Urrila, A.S., Hakkarainen, A., Castaneda, A., Paunio, T., Marttunen, M., Lundbom, N., 2017. Frontal Cortex Myo-Inositol Is Associated with Sleep and Depression in Adolescents: A Proton Magnetic Resonance Spectroscopy Study. Neuropsychobiology 75, 21–31. https://doi.org/10.1159/000478861
Wang, A.M., Pradhan, S., Coughlin, J.M., Trivedi, A., DuBois, S.L., Crawford, J.L., Sedlak, T.W., Nucifora, F.C., Nestadt, G., Nucifora, L.G., Schretlen, D.J., Sawa, A., Barker, P.B., 2019. Assessing Brain Metabolism With 7-T Proton Magnetic Resonance Spectroscopy in

Patients With First-Episode Psychosis. JAMA Psychiatry 76, 314–323. https://doi.org/10.1001/jamapsychiatry.2018.3637
Wijtenburg, S.A., Wright, S.N., Korenic, S.A., Gaston, F.E., Ndubuizu, N., Chiappelli, J., McMahon, R.P., Chen, H., Savransky, A., Du, X., Wang, D.J.J., Kochunov, P., Hong, L.E., Rowland, L.M., 2017. Altered Glutamate and Regional Cerebral Blood Flow Levels in Schizophrenia: A 1H-MRS and pCASL study. Neuropsychopharmacol. Off. Publ. Am. Coll. Neuropsychopharmacol. 42, 562–571. https://doi.org/10.1038/npp.2016.172
Wise, T., Taylor, M.J., Herane-Vives, A., Gammazza, A.M., Cappello, F., Lythgoe, D.J., Williams, S.C., Young, A.H., Cleare, A.J., Arnone, D., 2018. Glutamatergic hypofunction in medication-free major depression: Secondary effects of affective diagnosis and relationship to peripheral glutaminase. J. Affect. Disord. 234, 214–219. https://doi.org/10.1016/j.jad.2018.02.059
Xia, M., Wang, Junjie, Sheng, J., Tang, Y., Li, Chunyan, Lim, K., He, B., Li, Chunbo, Xu, Y., Wang, Jijun, 2018. Effect of Electroconvulsive Therapy on Medial Prefrontal γ-Aminobutyric Acid Among Schizophrenia Patients: A Proton Magnetic Resonance Spectroscopy Study. J. ECT 34, 227–232. https://doi.org/10.1097/YCT.0000000000000507
Yang, X.-R., Langevin, L.M., Jaworska, N., Kirton, A., Lebel, R.M., Harris, A.D., Jasaui, Y., Wilkes, T.C., Sembo, M., Swansburg, R., MacMaster, F.P., 2016. Proton spectroscopy study of the dorsolateral prefrontal cortex in youth with familial depression. Psychiatry Clin. Neurosci. 70, 269–277. https://doi.org/10.1111/pcn.12392
Yuksel, C., Du, F., Ravichandran, C., Goldbach, J.R., Thida, T., Lin, P., Dora, B., Gelda, J., O’Connor, L., Sehovic, S., Gruber, S., Ongur, D., Cohen, B.M., 2015. Abnormal high-energy phosphate molecule metabolism during regional brain activation in patients with bipolar disorder. Mol. Psychiatry 20, 1079–1084. https://doi.org/10.1038/mp.2015.13
Zhang, Xiaoliu, Tang, Y., Maletic-Savatic, M., Sheng, J., Zhang, Xuanhong, Zhu, Y., Zhang, T., Wang, Junjie, Tong, S., Wang, Jijun, Li, Y., 2016. Altered neuronal spontaneous activity correlates with glutamate concentration in medial prefrontal cortex of major depressed females: An fMRI-MRS study. J. Affect. Disord. 201, 153–161. https://doi.org/10.1016/j.jad.2016.05.014
Zong, X., Hu, M., Li, Z., Cao, H., He, Y., Liao, Y., Zhou, J., Sang, D., Zhao, H., Tang, J., Lv, L., Chen, X., 2015. N-acetylaspartate reduction in the medial prefrontal cortex following 8 weeks of risperidone treatment in first-episode drug-naïve schizophrenia patients. Sci. Rep. 5, 9109. https://doi.org/10.1038/srep09109
